# Health-related quality of life, psychological well-being and physical activity in patients with mild-to-moderate ascending aortic dilation - a cross-sectional case-control study

**DOI:** 10.1186/s12872-026-06616-9

**Published:** 2026-09-21

**Authors:** Fredrik Nilsson, Marcus Lindenberger, Sabina Borg, David Kylhammar, Petter Dyverfeldt, Filip Hammaréus, Lena Jonasson, Aleksandra Krzynska-Trzebiatowska, Lennart Nilsson, Chiara Trenti, Jan Engvall, Eva Swahn

**Affiliations:** 1 Department of Health, Medicine, and Caring Sciences, Linköping University, Linköping, Sweden; 2 Clinical Department of Cardiology in Linköping, Region Östergötland, Linköping, SE-581 85 Sweden; 3 Department of Health, Medicine and Caring Sciences, Division of Physiotherapy, Linköping University, Linköping, Sweden; 4 Department of Clinical Physiology in Linköping, Region Östergötland, Linköping, Sweden; 5 Division of Diagnostics and Specialist Medicine, Department of Health, Medicine and Caring Sciences, Linköping University, Linköping, Sweden; 6 Center for Medical Image Science and Visualisation, Linköping University, Linköping, Sweden; 7 Wallenberg Centre for Molecular Medicine, Linköping University, Linköping, Sweden; 8 Science for Life Laboratory, Linköping University, Linköping, Sweden

**Keywords:** Aneurysm, Ascending aorta, Quality of life, Depression, Anxiety, Exercise, Sedentary behaviour, Stress, psychological

## Abstract

**Aim:**

To investigate health-related quality of life (HRQoL), psychological well-being and physical activity in patients with mild-to-moderate ascending aortic dilation.

**Background:**

While ascending aortic dilation often require lifelong surveillance due to the increased risk of severe complications like aortic dissection or rupture, the impact of living with this known risk on patients’ psychological well-being and overall quality of life remains less studied.

**Methods:**

Subjects with ascending aortic dilation ≥ 40 mm and age- and sex-matched controls were identified in the general population-based Swedish CArdioPulmonary bioImage Study (SCAPIS) in Linköping (*n* = 5053). Of these, 67 cases and 50 controls completed the questionnaires. HRQoL, psychological well-being, physical activity and capacity were assessed using validated forms. Group comparisons were made with independent t-tests or Mann-Whitney U. For correlation analyses, Spearman’s rank correlation was used.

**Results:**

No statistically significant differences in quality of life were observed between cases and controls. Anxiety and depression scores were likewise comparable across groups (*p* = 0.228 and 0.307, respectively). Perceived stress (*p* = 0.096), physical capacity (*p* = 0.173), and physical activity (*p* = 0.471) also did not differ significantly between cases and controls.

With respect to sedentary behaviour, cases demonstrated a higher mean rank than controls (62 vs. 52), although the p-value marginally exceeded the conventional threshold for significance (*p* = 0.062). Finally, no significant correlations were identified between aortic diameter and perceived stress, anxiety, or depression.

**Conclusions:**

In this population-based cohort, no statistically significant differences in HRQoL, psychological well-being, and physical activity were observed between subjects with mild to moderate ascending aortic dilation and matched controls. However, given the small sample size, small differences between groups cannot be excluded. Balanced management strategies that promote safe physical activity, psychological well-being and HRQoL remain important in this population.

**Graphical Abstract:**

**Figure:**
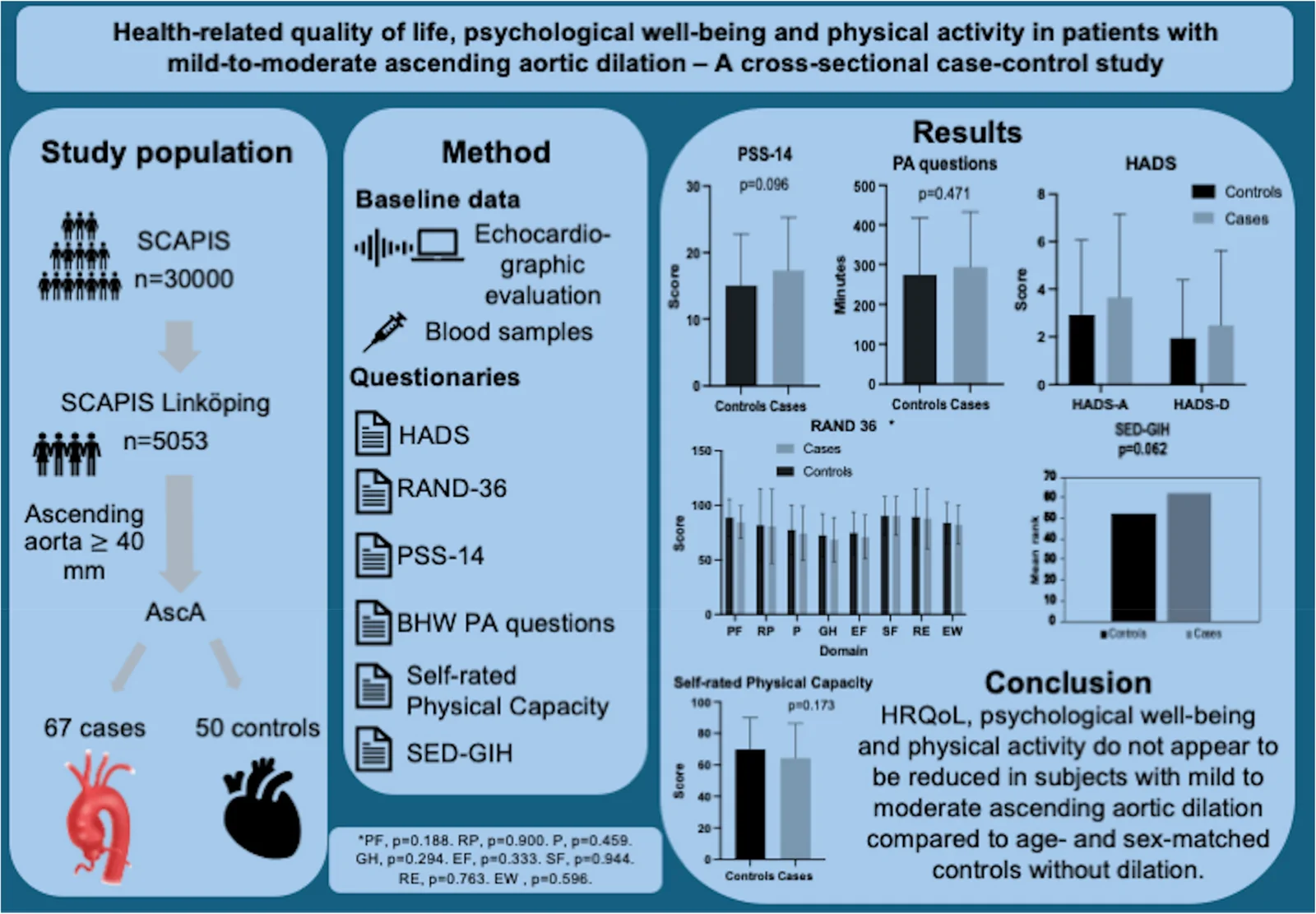
SCAPIS = Swedish CArdioPulmonary bioImage study; AscA = ASCending Aorta study; HADS = hospital anxiety and depression scale; HADS-A = hospital anxiety and depression scale – anxiety; HADS-D = hospital anxiety and depression scale – depression; PSS-14 = perceived stress scale-14; BHW PA questions = Board of health and welfare physical activity; SED-GIH = sedentary behaviour-GIH; HRQoL = health-related quality of life; PF = physical functioning; RP = role limitations due to physical health problems; P = bodily pain; GH = general health perceptions; EF = energy/fatigue; SF = social functioning; RE = role limitations due to emotional problems; EW = emotional well-being.

## Introduction

Ascending aortic dilation, a disease characterized by abnormal enlargement of the aorta, poses significant medical concerns due to the risk of severe complications like aortic dissection and rupture [1]. These patients require regular monitoring and, in some cases, prophylactic surgical intervention when reaching specific diameters or if the progression rate is fast [2]. Advancements in medical imaging have led to an increased detection of incidental aortic dilations, most of which are mild to moderate but still require follow-up. Even less pronounced dilations are associated with an increased risk of dissection compared with normal aortic diameter [3]. Patients may be under surveillance for decades and live long with knowledge of the increased risk for an aortic event. Beyond the risk of aortic complications, the broader impact on patients’ psychological well-being and overall quality of life remains less studied. Exploring these questions is essential to understand the consequences of living with dilation of the ascending aorta.

Several studies have examined health-related quality of life (HRQoL) after surgical repair of a thoracic aneurysm and have reported similar HRQoL as in the general population [4, 5]. However, studies on HRQoL in conservatively managed patients with ascending aortic dilation are sparse. Olsson and Franco-Cereceda studied HRQoL in 178 patients with thoracic aortic disease from an aortic outpatient clinic. Using Short form-36, which is a validated form assessing HRQoL, they found a negative impact in three out of eight domains (physical functioning, role physical and general health) compared to a reference group, although the differences were small [6]. Another large meta-study, mainly involving descending aortic aneurysms, showed a decrease in HRQoL with increased age and smoking, but no association with aneurysm size [7]. Assessing quality of life in patients managed conservatively is important, since it is one of several factors that may influence shared decision-making regarding timing of prophylactic surgery [2, 8].

A bidirectional relationship between cardiovascular disease (CVD) and depression has been widely reported in the literature and patients with CVD and concomitant depression have a worse outcome than patients with CVD alone [9, 10]. Moreover, patients with symptoms of depression exhibit a higher risk of developing abdominal aortic aneurysm than those without [11]. In contrast, research regarding anxiety in patients with ascending aortic dilation is sparse. Benke et al. found increased levels of anxiety in everyday life in patients with Marfan syndrome scheduled for surgery due to aortic dissection compared to healthy individuals [12]. These studies indicated a potential association between psychological well-being and aortic disease, a relationship that warrants further investigation, particularly in mild to moderate ascending aortic dilation.

Patients with aortic dilation are often advised to limit certain physical exercise to prevent aortic events [13]. However, current guidelines are not clear on how and when to implement such restrictions, and they are mainly for patients with wider aneurysms. The implications of such limitations are previously described by McEntire et al., indicating increased psychological distress following activity restrictions in patients with ascending aortic dilation [14]. Despite this, physical inactivity and sedentary behavior remain well-known modifiable risk factors for CVD and CVD mortality [15]. A recent prospective cohort study showed a protective association between physical activity and the development of aortic aneurysms, particularly abdominal aortic aneurysms, whereas the association for thoracic aortic aneurysms was weaker and less consistent [16].

The aim of the current study was to investigate HRQoL, psychological well-being and physical activity in patients with mild to moderate ascending aortic dilation.

## Methods

### Study population

**Fig. 1:**
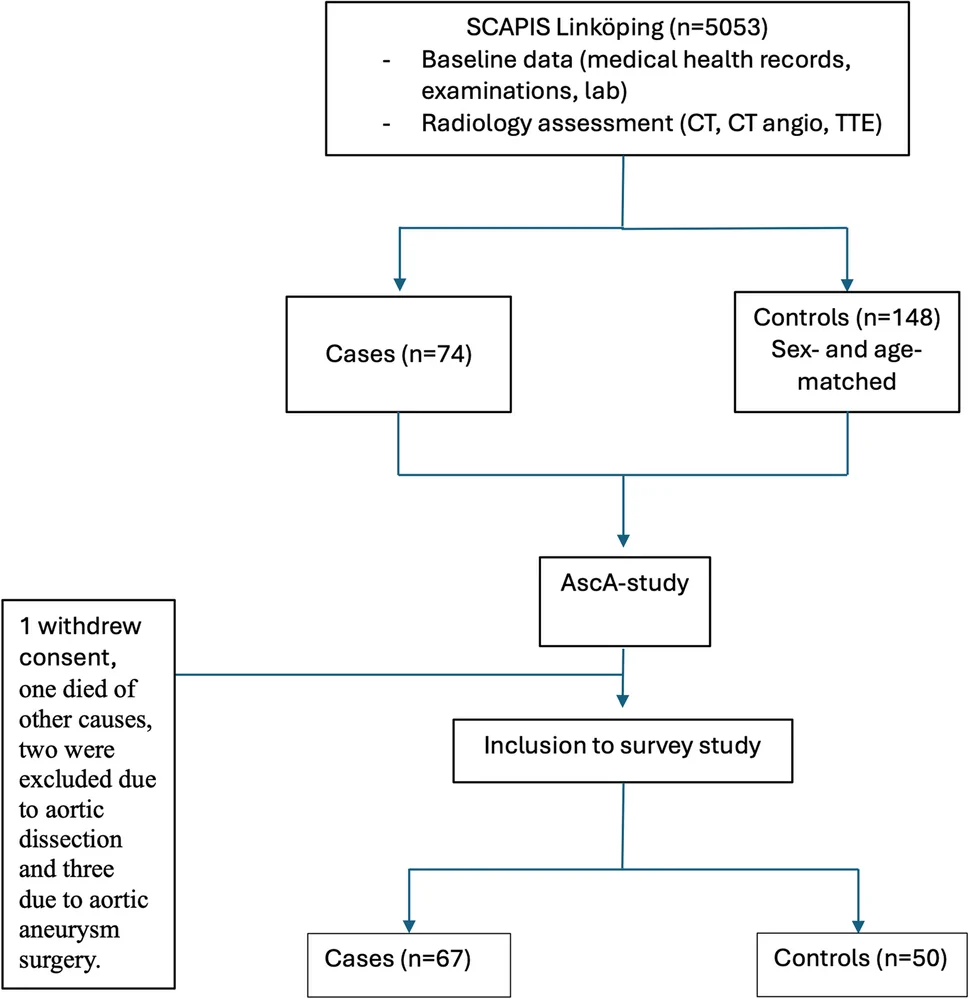
Flow chart. SCAPIS = Swedish CArdioPulmonary bioImage Study; CT = computed tomography; TTE = transthoracic echocardiography; AscA = Ascending Aorta Study

The study population was derived from the Swedish CArdioPulmonary bioImage study (SCAPIS) [17]. In SCAPIS, 30 000 men and women aged 50–65 were randomly selected from the general population and 5053 of these were included in Linköping. Among the 5053 SCAPIS participants in Linköping, 74 individuals (2%) were found to have an aortic root and/or ascending aortic diameter ≥ 40 mm on computed coronary tomography angiography (CCTA), non-contrast thoracic CT, or transthoracic echocardiography (TTE), measured according to guidelines at the time of inclusion. These individuals were included in the Ascending Aorta (AscA) study. Based upon age and sex, two matched controls for each subject from the same SCAPIS Linköping cohort (diameter < 40 mm) were included (*n* = 148). All cases and controls from the AscA-study were asked to participate in the present survey study, this was approximately 5 years after their aortic dilation diagnosis. Seventy-four cases were included in the original AscA study. However, during follow-up, one participant withdrew consent, one died of other causes, two were excluded due to aortic dissection and three due to aortic aneurysm surgery, and the remaining 67 subjects were eligible for the present study. Fifty controls (34%) were enrolled in the study. Because of the limited number of participating controls, 16 controls were matched to two cases each. (Fig. 1, flow chart).

### Ethics

All study subjects gave written informed consent. The study adheres to the Declaration of Helsinki and was ethically approved (Dnr. 2018/24–31 and 2023-02266-02).

### Patient and public involvement

Patients or the public were not involved in the design, or conduct, or reporting, or dissemination plans of our research.

### Imaging and anthropometric data

TTE was performed to evaluate the diameter of the ascending aorta during follow-up. The aortic diameter was measured in the parasternal long-axis view using the leading edge-to-leading edge method, in accordance with current guidelines [18]. Blood samples, blood pressure and other anthropometric data were collected from the original SCAPIS study, as previously described [17, 19]. Since the inclusion of the AscA-population started 2019 and the questionnaires were completed in 2023/2024, ascending aortic diameter from echocardiographic measurements under 2023/2024 were also presented for the cases.

### Questionnaires

To assess psychological well-being, including HRQoL, three forms were used: RAND-36; Hospital Anxiety and Depression Scale (HADS) and Perceived Stress Scale (PSS-14). All in Swedish versions.

RAND-36 is a free and equivalent version of the widely used SF-36 to assess HRQoL [20–22]. The version used, RAND-36-Item Health Survey 1.0, concerns eight health concepts: physical functioning, bodily pain, role limitations due to physical health problems, role limitations due to personal or emotional problems, emotional well-being, social functioning, energy/fatigue, and general health perceptions. Each domain is graded from 0 to 100, where a higher score represents better health.

HADS is a widely used and validated 14-item questionnaire designed to assess symptoms of anxiety and depression [23–25]. The instrument consists of two subscales: HADS-Anxiety (HADS-A) and HADS-Depression (HADS-D), each consisting of seven items. Each item generates 0–3 points, with the points from each domain (anxiety or depression) added together when interpreting results; as such, scores range from 0 to 21, where 0–7 is considered normal, 8–10 indicate possible clinically relevant symptoms and 11 or above indicate probable clinically relevant symptoms. HADS was analysed for anxiety and depression separately.

PSS-14 evaluates stress over the past month. PSS-14 has previously been translated to Swedish by Eskin et al. with good reliability and internal consistency [26, 27]. The questionnaire has 14 items, each with a Likert scale from 0 (never) to 4 (very often), scores range from 0 to 56, and a score of 25 or higher indicates increased stress levels.

To evaluate physical capacity the form Self-rated Physical Capacity, visual analogue scale was used. The patient rates the worst possible physical capacity (0) to the best possible physical capacity (100). Two questions recommended by the Swedish National Board of Health and Welfare (BHW PA questions) for evaluation of the level of physical activity were used. Question one concerns high-intensity exercise in six scales from 0 to > 120 min during a regular week. Question two addresses everyday physical activity (moderate intensity) in seven scales from 0 to > 300 min during a regular week. Scores from question one and two are then added to obtain Activity minutes [28–32]. Sedentary behaviour was evaluated using a question (SED-GIH) in seven scales regarding time spent sitting during a usual day (1 = virtually all day to 7 = never).

### Statistics

Group comparisons were made with independent t-tests for all continuous quantitative variables and for all forms except for SED-GIH sedentary behaviour, where the nonparametric Mann-Whitney U test was used since the possible answers were on an ordinal scale. For correlation analyses, Spearman’s rank correlation was used. A p-value < 0.05 was considered statistically significant. A few subjects for each form were excluded during analysis due to missing data. Since the missing data were not assumed to be random and the data loss was minimal, multiple imputation was not used. Analyses were performed using SPSS, version 29 (IBM, Armonk, NY, USA).

## Results

### Baseline characteristics

The study population consisted of 67 cases and 50 controls with mean age 59 ± 4 years and a predominance of men (80%) among the cases. Baseline characteristics are presented in Table 1 and the number of respondents for each questionnaire is presented in Table 2.

**Table 1:** Baseline characteristics

	Cases, *n* = 67	Controls, *n* = 50	*P*
Age, mean (SD), years	59 ± 4	59 ± 4	0.951
Male sex, n (%)	53 (79)	40 (80)	0.906
BMI, mean (SD), kg/m2	28 ± 4	27 ± 4	0.094
Smoking, n (%)	5 (7)	5 (10)	0.627
Diabetes, n (%)	6 (10)	4 (8)	0.802
COPD, n (%)	2 (3)	3 (6)	0.454
Asthma, n (%)	6 (10)	1 (2)	0.104
Inflammatory disease, n (%)	8 (12)	5 (10)	0.741
Coronary artery disease, n (%)	2 (3)	5 (10)	0.127
CT Aortic diameter at inclusion, mean (SD), mm	43 ± 3	34 ± 3	< 0.001*
Latest echocardiographic ascending aortic diameter, mean (SD), mm	45 ± 3	N/A	N/A

Inflammatory disease; rheumatoid arthritis, psoriasis, inflammatory bowel disease, other systemic inflammatory disease. CT, computed tomography

*SD* Standard deviation, *BMI* Body mass index, *COPD* Chronic obstructive pulmonary disease

*Statistical significance. Latest echocardiographic ascending aortic diameter was only collected for cases

**Table 2:** Group comparison for each questionnaire

	Cases Mean ± SD (respondents)	Control Mean ± SD (respondents)	Mean difference (95%CI)	*P*
RAND 36				
Physical functioning	85 ± 15 (67/67)	89 ± 17 (48/50)	-4.1(-10,1 to 2.0)	0.188
Role limitations due to physical health	81 ± 34 (67/67)	82 ± 34 (48/50)	-0.8(-13.5 to 11.8)	0.900
Role limitations due to emotional problems	88 ± 28 (67/67)	90 ± 26 (48/50)	-1.5(-11.5 to 8.5)	0.763
Energy/fatigue	71 ± 20 (65/67)	75 ± 19 (48/50)	-3.6(-11.1 to 3.8)	0.333
Emotional well-being	82 ± 18 (65/67)	84 ± 19 (48/50)	-1.8(-8.7 to 5.0)	0.596
Social functioning	90 ± 18 (66/67)	90 ± 18 (48/50)	-0.02(-6.7 to 6.6)	0.994
Pain	74 ± 25 (66/67)	78 ± 22 (48/50)	-3.3(-12.3 to 5.6)	0.459
General health	69 ± 20 (67/67)	73 ± 20 (48/50)	-4.0(-11.5 to 3.5)	0.294
HADS				
Anxiety	3.7 ± 3.5 (65/67)	2.9 ± 3.1 (50/50)	0.76(-0.48 to 1.99)	0.228
Depression	2.5 ± 3.2 (65/67)	1.9 ± 2. (50/50)	0.54 (-0.50 to 1.57)	0.307
PSS-14	17 ± 8 (66/67)	15 ± 8 (50/50)	2.47 (-0.45 to 5.39)	0.096
Physical activity				
Self-rated Physical Capacity, visual analogue scale	64 ± 22 (63/67)	70 ± 20 (49/50)	-5.4 (-13 to 2.5)	0.173
PA questions, (activity minutes)	294 ± 139 (61/67)	274 ± 143 (49/50)	20 (-34 to 74)	0.471

*SD* Standard Deviation, *CI* Confidence Interval, *HADS* Hospital Anxiety Depression Scale, *PSS* Perceived Stress Scale, *PA* Physical activity

### HRQoL

**Fig. 2:**
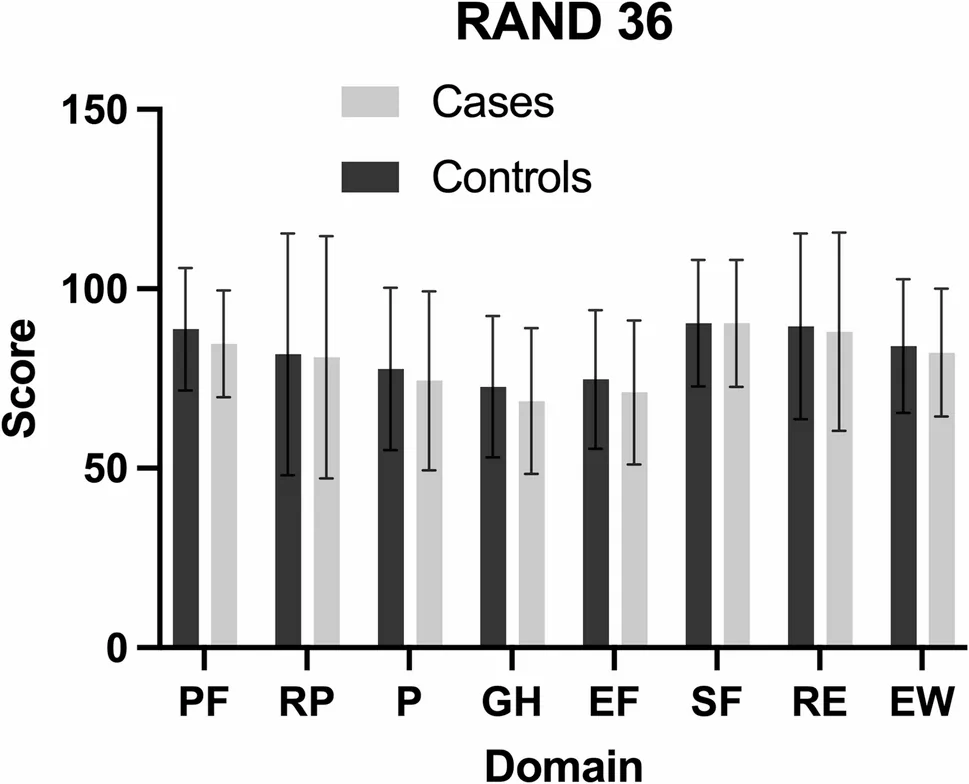
Mean scores for all eight domains of RAND 36 between cases and controls. PF = physical functioning, *p* = 0.188. RP = role limitations due to physical health problems, *p* = 0.900. P = bodily pain, *p* = 0.459. GH = general health perceptions, *p* = 0.294. EF = energy/fatigue, *p* = 0.333. SF = social functioning, *p* = 0.944. RE = role limitations due to emotional problems, *p* = 0.763. EW = emotional well-being, *p* = 0.596

The RAND-36 survey showed lower mean scores for cases compared to controls for all domains (Table 2; Fig. 2). However, none of these were significant. The most pronounced difference was seen in Physical Functioning, with a mean score of 85 ± 15 among cases compared to 89 ± 17 among controls, although not significant (*p* = 0.188).

### Psychological well-being

**Fig. 3:**
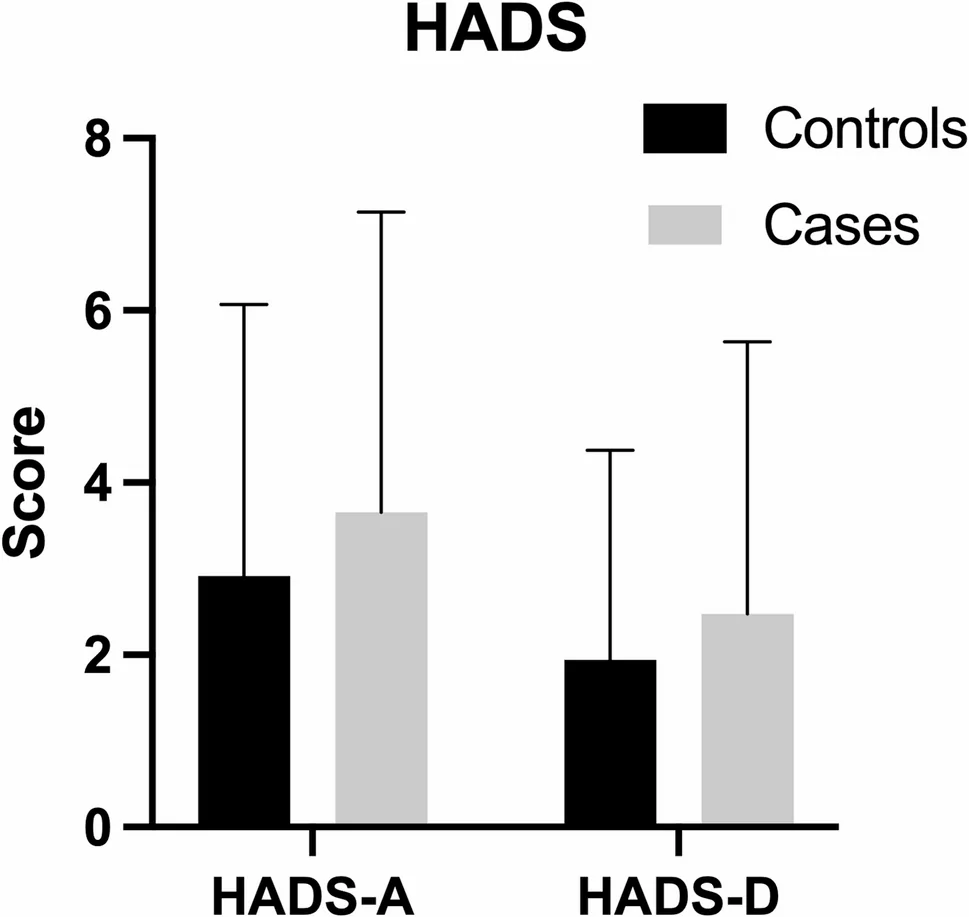
Mean scores for cases and controls for HADS. HADS-A = hospital anxiety and depression scale – anxiety. P=0.228. HADS-D = hospital anxiety and depression scale – depression. p=0.307

**Fig. 4:**
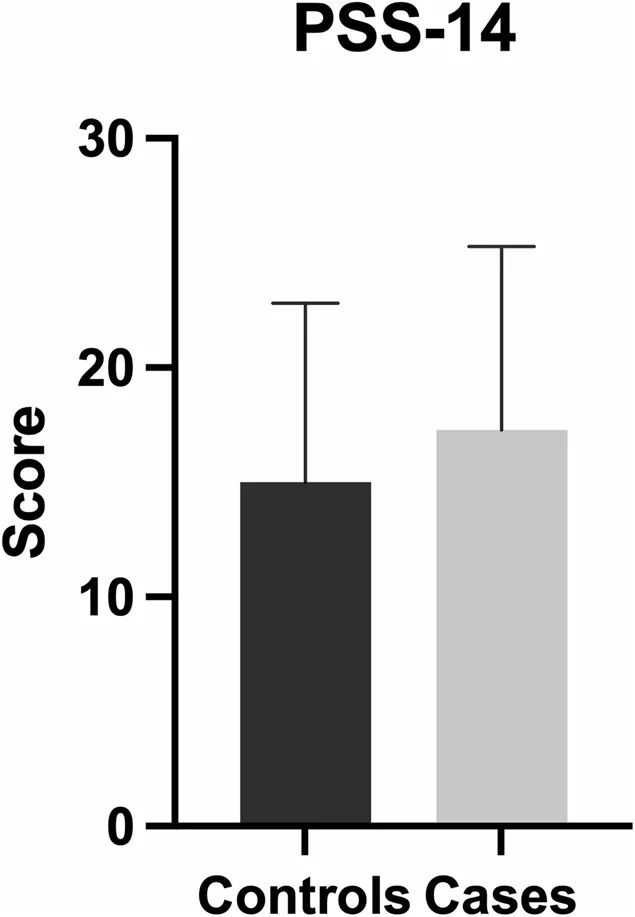
Mean scores for cases and controls for PSS-14. PSS-14 = Perceived stress scale-14. *p* = 0.096

For anxiety, mean score was 3.7 ± 3.5 among cases and 2.9 ± 3.1 for controls, with a mean difference of 0.76 (-0.48 to 1.99, *p* = 0.228). For depression, mean score was 2.5 ± 3.2 for cases and 1.9 ± 2.4 for controls, mean difference of 0.54 (-0.50 to 1.57, *p* = 0.307). For PSS-14 the mean score was 17 ± 8 and 15 ± 8 for cases and controls respectively, no significant differences between groups (Table 2; Figs. 3 and 4). Correlation analyses between echocardiographic aortic diameter and PSS-14, HADS-D and HADS-A showed no significant correlation (Table 3).

**Table 3:** Spearman''s rank correlation coefficients (ρ) between aortic diameter and psychological variables among cases

Variable	Spearman’s ρ	*p*-value	*n*
HADS Anxiety	0.176	0.169	63
HADS Depression	-0.036	0.779	63
PSS-14	0.036	0.778	64

Aortic diameter, latest ascending aortic diameter from echocardiography, measured leading edge- leading edge

*HADS* Hospital anxiety depression scale, *PSS* Perceived stress scale

### Physical activity

**Fig. 5:**
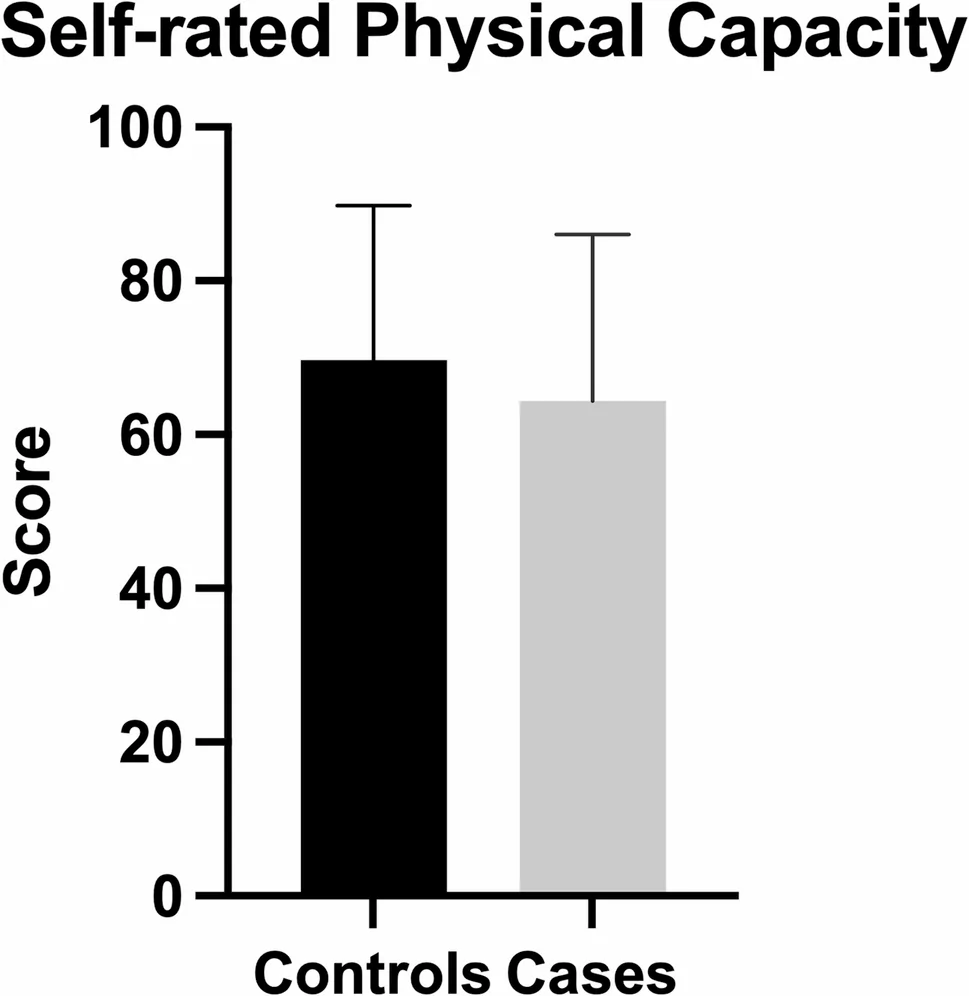
Mean scores for cases and controls for Self-rated Physical Capacity, visual analogue scale. *p* = 0.173

**Fig. 6:**
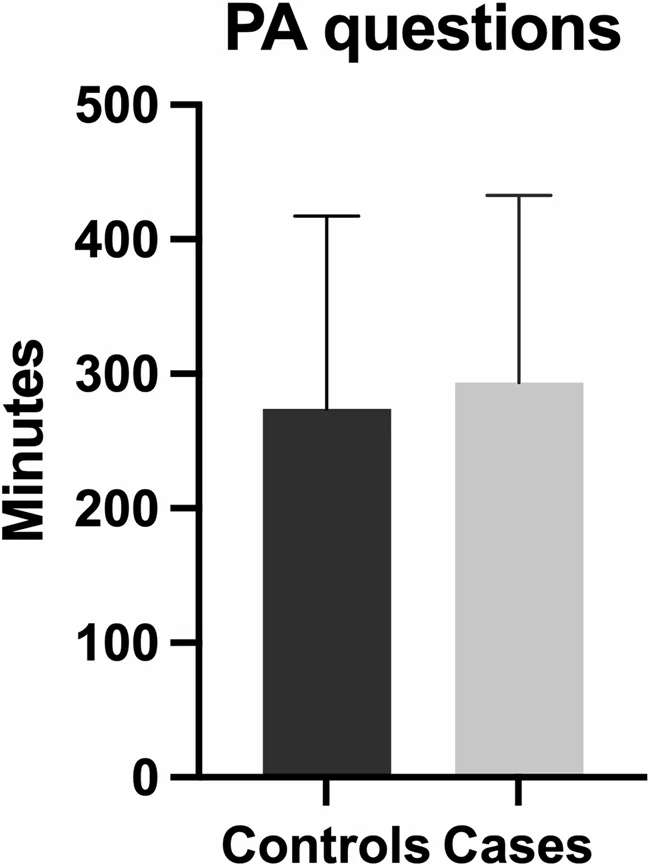
Mean scores for cases and controls for PA-questions. PA-questions (activity minutes) = Physical activity questions. *p* = 0.471

**Fig. 7:**
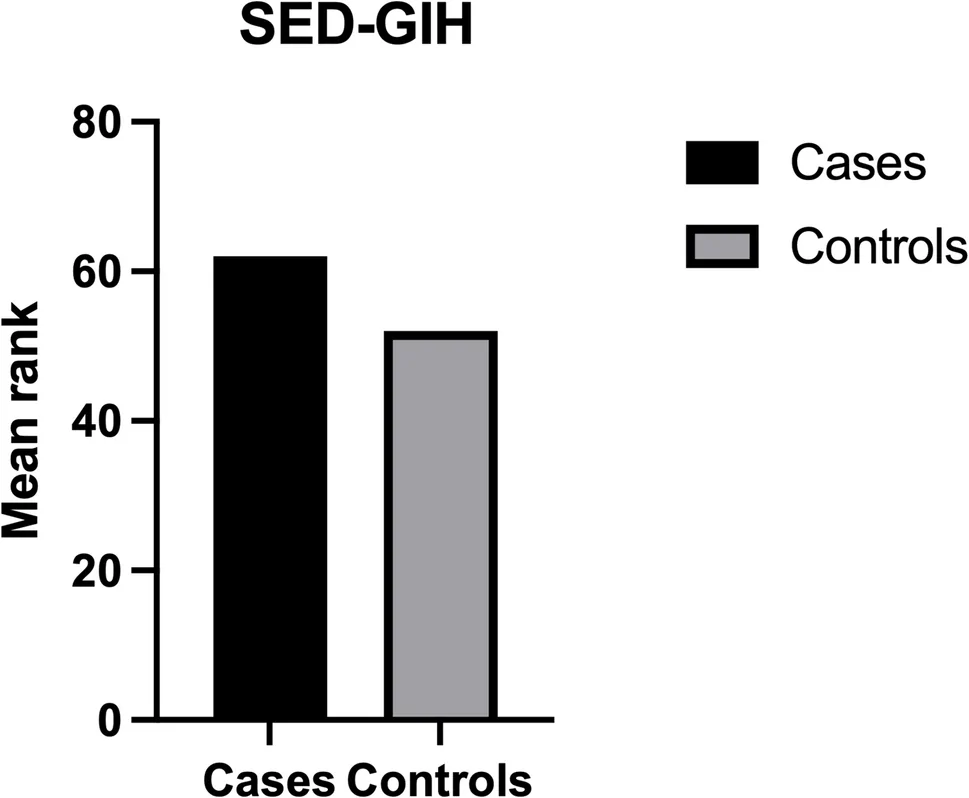
Mean rank for cases and controls for SED-GIH. SED-GIH = Sedentary behavior-GIH. *p* = 0.06

Self-rated physical capacity, visual analogue scale showed a mean score of 64 ± 22 for cases and 70 ± 20 for controls, mean difference − 5.4 (-13 to 2.5, *p* = 0.173), (Fig. 5). For BHW PA questions (activity minutes) cases reported a mean of 294 ± 139 and controls 274 ± 143, mean difference 20 (-34 to 74 *p* = 0.471), (Fig. 6). Lastly, for SED-GIH sedentary behaviour, cases had a mean rank of 62 compared to 52 among controls, with a p-value just above the level of significance of 0.062 between groups (Fig. 7).

## Discussion

This study provides new insights into HRQoL, psychological well-being and physical activity in patients with mild to moderate ascending aortic dilation. Our results suggest that patients with mild to moderate ascending aortic dilation generally do not exhibit significant differences in HRQoL, psychological well-being or physical activity compared to matched controls.

Olsson and Franco-Cereceda (2013) assessed patients with chronic thoracic aortic disease, using Short Form 36 (SF-36) which is comparable with RAND-36, and found a small but significant reduction in several domains of the SF-36 compared to a healthy cohort [6]. They studied a heterogenous group, with aortic diameter not specified, with one third affected by aortic dissection. Furthermore, dyspnea appeared as the single most prevalent predictor for reduced SF-36, a symptom not associated with aortic aneurysms of small to moderate size.

Previous studies have shown a clear association between chronic diseases and HRQoL, where reduced HRQoL were driven by severe specific symptoms [33]. We studied mild to moderate dilation with no physical symptoms, but rather, if any, psychological effect. We believe that this to a substantial part explains why we did not observe any differences between the groups.

We found no association between mild to moderate aortic dilation and depression in our study, even though a strong association between CVD and depression is well established from previous studies [9, 10]. The same findings were true for perceived stress and anxiety. CVD in general often causes symptoms and require specific treatment as surgery and/or medical treatment. However, this was not true for our cohort, which probably represents the primary explanation to our results. A cohort with wider diameter, regular follow up by a cardiologist and with a more extensive health care need might show a different outcome in these parameters. Correlation analyses showed no correlation between psychological well-being and aortic diameter in our cohort, which is in line with the results when comparing cases and controls as groups.

No differences were found between cases and controls in parameters regarding physical activity. Our cohort is derived from the general population and consist of individuals with asymptomatic mild to moderate dilation where the majority have not been subjected to activity restrictions which probably explains the lack of difference between cases and controls in these parameters.

The 2020 ESC guidelines on sports cardiology and exercise in patients with CVD provide recommendations for exercise in patients with aortic aneurysms [13]. However, these recommendations are labelled as class IIa or IIb and are based on level C evidence. There is a risk that these recommendations overestimate the risk with exercise while underestimating the risk of sedentary behavior.

McEntire et al. studied 79 patients with thoracic aneurysms and/or aortic dissection, all of whom had received activity restrictions, and reported that some individuals experienced increased psychological distress as a result [14]. Furthermore, a qualitative study published 2021 by Velvin et al. on patients with hereditary thoracic aortic disease identified several factors contributing to a sedentary lifestyle, including inconsistent professional advice, non-engaging activities, unpredictable health conditions and fear of exercise [34]. These factors will also be relevant to our study population, despite not being fully reflected in the physical activity outcomes even though the results for sedentary behavior almost met the level of significance for the non-parametric test when cases and controls were compared. It is of great value to carefully balance physical activity-restrictions with the risks associated with inactivity in these patients. When recommending physical activity restrictions to mitigate the risk of aortic dissection or rupture, it is essential to simultaneously encourage safe and appropriate physical activity to reduce the risks accompanied with sedentary behavior. It is worth noting that subjective methods for quantifying sedentary behavior, as used in this study, are known to underestimate actual sedentary time ^33^. However, such inaccuracies are likely to affect both groups equally, minimizing their impact on between-groups comparisons.

Future research is needed to fully understand the psychological aspect of aortic dilation. Differences might be easier to find in a larger population with wider diameters. Furthermore, a qualitative approach could reveal more insight about the relation between aortic dilation and physical activity and the impact of activity restrictions.

### Limitations

A few limitations need to be acknowledged in this study. Self-reported questionnaires may be subject to biases, such as recall bias or social-desirability bias. A more objective evaluation of physical activity, for example using wearable activity monitors, could provide a more accurate picture. To minimize the impact of confounders, all cases were matched based on age and sex to a control. However, due to the relatively small sample size, further matching was not feasible. Since the control group was smaller than initially intended, a few controls served as controls for two cases, which all together may have limited the statistical power of the study. In addition, the relatively low participation rate among invited controls may have introduced selection bias, as participants could have differed from non-participants in ways not captured by the variables assessed in this study. Lastly, according to guidelines, we used 40 mm as cut off for inclusion without adjusting for body surface area and the normal variations between sexes. As a result, our sample included disproportionately more men.

## Previous presentations

The baseline characteristics have previously been described by Swahn E et al., Prevalence and determinants of dilated ascending aorta in a Swedish population: a case-control study. DOI: https://doi.org/10.1093/ehjopen/oead085.

Echocardiographic data has previously been described by Kylhammar et al., Follow-up of incidentally detected mild to moderate ascending aortic dilation and risk factors for rapid progression in a Swedish middle-aged population. DOI: https://doi.org/10.1136/heartjnl-2024-325409.

None of the other data presented in this study has previously been published.

## Conclusion

In this population-based cohort, no statistically significant differences in HRQoL, psychological well-being and physical activity were observed between subjects with mild to moderate ascending aortic dilation compared to age- and sex-matched controls without dilation. Given the relatively small sample size, small differences between groups cannot be excluded. However, since these patients often are under years of surveillance and aortic dilation frequently is a progressive disease, it is essential to practise balanced management strategies that emphasise both safe physical activity, psychological well-being and HRQoL.

## Data Availability

The datasets used and/or analysed during the current study are available from the corresponding author upon reasonable request. Contact: fredrik.nilsson@liu.se.

## Abbreviations

HRQoL: Health-related quality of life
SCAPIS: Swedish CArdioPulmonary bioImage Study
CVD: Cardiovascular disease
CCTA: Computed coronary tomography angiography
TTE: Transthoracic echocardiography
HADS: Hospital Anxiety and Depression Scale
PSS: 14-Perceived Stress Scale
HADS: A-HADS-Anxiety
HADS: D-HADS-Depression
CT: Computed tomography
AscA: Ascending Aorta Study
PF: Physical functioning
RP: Role limitations due to physical health problems
P: Bodily pain
GH: General health perceptions
EF: Energy/fatigue
SF: Social functioning
RE: Role limitations due to emotional problems
EW: Emotional well-being
SED: GIH-Sedentary behavior-GIH
SD: Standard deviation
BMI: Body mass index
COPD: Chronic obstructive pulmonary disease
CI: Confidence Interval
